# SMAC Mimetic BV6 Enables Sensitization of Resistant Tumor Cells but also Affects Cytokine-Induced Killer (CIK) Cells: A Potential Challenge for Combination Therapy

**DOI:** 10.3389/fped.2014.00075

**Published:** 2014-07-18

**Authors:** Eva Rettinger, Andreas Glatthaar, Behnaz Ahangarian Abhari, Sarah Oelsner, Verena Pfirrmann, Sabine Huenecke, Selim Kuçi, Hermann Kreyenberg, Andre M. Willasch, Thomas Klingebiel, Simone Fulda, Peter Bader

**Affiliations:** ^1^Division for Stem Cell Transplantation and Immunology, Department for Children and Adolescents Medicine, University Hospital Frankfurt, Goethe University Frankfurt am Main, Frankfurt, Germany; ^2^Institute for Experimental Cancer Research in Pediatrics, Goethe University Frankfurt am Main, Frankfurt, Germany; ^3^Georg-Speyer-Haus, Institute for Biomedical Research, Frankfurt, Germany

**Keywords:** BV6, cellular therapy, CIK cells, leukemia, tumors

## Abstract

Allogeneic hematopoietic stem cell transplantation (HSCT) is an established treatment option for high-risk hematological malignancies, and may also be offered to patients with solid malignancies refractory to conventional therapies. In case of patients’ relapse, refractory tumor cells may then be targeted by cellular therapy-based combination strategies. Here, we investigated the potential of small molecule IAP (SMAC mimetic) BV6 in increasing cytokine-induced killer (CIK) cell-mediated cytotoxicity against different tumor targets. Four-hour pre-incubation with 2.5 μMol BV6 moderately enhanced CIK cell-mediated lysis of hematological (H9, THP-1, and Tanoue) and solid malignancies (RH1, RH30, and TE671). However, BV6 also increased apoptosis of non-malignant cells like peripheral blood mononuclear cells and most notably had an inhibitory effect on immune cells potentially limiting their cytotoxic potential. Hence, cytotoxicity increased in a dose-dependent manner when BV6 was removed before CIK cells were added to tumor targets. However, cytotoxic potential was not further increasable by extending BV6 pre-incubation period of target cells from 4 to 12 h. Molecular studies revealed that BV6 sensitization of target cells involved activation of caspases. Here, we provide evidence that SMAC mimetic may sensitize targets cells for CIK cell-induced cell death. However, BV6 also increased apoptosis of non-malignant cells like CIK cells and peripheral mononuclear cells. These findings may therefore be important for cell- and small molecule IAP-based combination therapies of resistant cancers after allogeneic HSCT.

## Introduction

Allogeneic hematopoietic stem cell transplantation (HSCT) is an established strategy for treatment of high-risk hematological malignancies, and is also offered to patients with solid malignancies refractory to conventional therapies (1). But still, due to tumor cell resistance, the therapeutic success of this approach is limited by patients’ relapse. Hence, new therapeutic strategies are needed, which may combine cellular therapies and small molecule drugs for retargeting of resistant tumor cells. Immune cell-based combination strategies may not only include chimeric antigen receptor (CAR) bearing T cells (2, 3), T cell receptor (TCR) transduced T cells (4, 5), or specific cytotoxic T lymphocyte (CTL) clones (6, 7), but may also include non-specific immune cell approaches using cytokine-induced killer (CIK) cells.

CIK cells are activated and expanded *in vitro* from peripheral blood mononuclear cells (PMNCs) by timed addition of cytokines. Expanded CIK cells represent a heterogeneous population of CD3^+^CD56^−^ T cells and CD3^−^CD56^+^ natural killer (NK) cells. T cells in part share both CD3^+^ T cell and CD56^+^ NK cell phenotype (CD3^+^CD56^+^ T-NK cells). CIK cells are able to eradicate a variety of hematological and solid malignancies in a non-major histocompatibility complex (MHC)-restricted manner without possessing significant alloreactive potential (8–17). Therefore, the application of CIK cells has evolved from experimental observations into early clinical allogeneic HSCT studies. These trials included transplanted patients who had relapsed from hematological malignancies. Most of these patients showed transient clinical responses after CIK cell infusions (18–20). Anti-leukemic activity of CIK cells, without long lasting efficacy, may suggest limited lifespan of infused CIK cells or resistance mechanisms developed by target cells.

Inhibitors of apoptosis (IAP) proteins are associated with chemo-resistance, disease progression, and poor prognosis in different cancers (21, 22). Therefore, IAPs may be interesting for retargeting tumor cells toward unspecific CIK cell-based killing by using a combination of CIK cells and small molecule IAP (SMAC mimetics/IAP antagonists) (23).

The divergent structures of SMAC mimetics originate from the conserved AVPI tetrapeptide N-terminal sequence of SMAC/DIABLO (direct inhibitor of apoptosis-binding protein with low isoelectric point/direct IAP-binding protein with low pI) that binds to the BIR domains of IAP proteins with high affinities to promote cell death and inhibit tumor growth in *in vivo* models. In response to apoptotic stimuli, following the death trigger, mitochondria may become selectively permeabilized, SMAC along with pro-apoptotic proteins such as cytochrome *c* are released from the intermembrane space of mitochondria into the cytoplasm (24). SMAC is a dimer and interacts with its four N-terminal amino acid residues (AVPI) with XIAP to abrogate XIAP-mediated inhibition of caspases-3 and -9 leading to dissociation of bound caspases from XIAP (25).

One important contribution of IAP proteins to cell survival and tumorigenesis is the ability of several IAP proteins to regulate alternative nuclear factor (NFκB) signaling. cIAP1 and cIAP2 are involved in degradation of the MAP3 kinase, NFB inducing kinase (NIK) in the NFκB pathway (23, 26–29), and contribute to activation of the classical NFκB pathway by tumor necrosis factor (TNF) stimulation (30–32). Besides preventing the XIAP interaction with caspases, SMAC mimetics induce activation of the NFκB pathway by binding to cIAP1 and cIAP2 and stimulating the E3 ubiquitin-ligase activity of the cIAP proteins (33).

So far, several small molecules that mimic the IAP binding of motif of SMAC and pharmacologically inhibit IAP protein function were designed and described (34). Beside BV6 (23), birinapant (TL32711) a biindole-based bivalent SMAC mimetic recently showed promising synergistic cytotoxicity of several widely used anti-cancer agents in pre-clinical analyses (35, 36).

This study was performed to assess the role of bivalent SMAC mimetic BV6 in increasing susceptibility of target cells toward CIK cell-mediated killing in cell line models. Our findings may be important for cell-based combination strategies in the treatment of resistant tumor cells.

## Materials and Methods

### Cell lines

T cell lymphoma cell line H9, subtype M4 acute myeloid leukemia cell line THP-1, precursor-B acute lymphoblastic leukemia cell line Tanoue, Ewing sarcoma cell line RH1, alveolar rhabdomyosarcoma cell line RH30, and embryonal rhabdomyosarcoma cell line TE671 were obtained and cultured as previously described (13, 17).

### CIK cells

CIK cells were generated as previously described from PMNCs of healthy donors in accordance with the institutional review board approval and after written informed consent (Geschäfts-Nr.: 298/07) (17). In brief, after 10 days of *in vitro* stimulation with interferon (IFN)-γ, anti-CD3 antibody, and interleukin (IL)-2 and IL-15, CIK cells were harvested and used in experiments.

### Europium release assay

Europium release assay was used to assess lytic activity of CIK cells with or without pre-treatment of target cells with SMAC mimetic BV6. In brief, target cells used (H9, THP-1, Tanoue, RH1, RH30, TE671, and PMNCs) were labeled with BATDA (DELFIA^®^ BATDA Labeling Reagent, PerkinElmer, Waltham, MA, USA). Labeled target cells were pre-incubated with 2.5 μMol BV6 for 4 h or remained without BV6 treatment. To further analyze dosage and timing of target cell sensitization by BV6, selected hematological and solid malignancies (THP-1 and RH30) were labeled with BATDA, sensitized with 2.5 and 10 μMol BV6 for 4 h and were washed to remove BV6 before being used in experiments. In addition, more resistant targets cell lines (RH30 and TE671) were pre-incubated with 2.5 and 10 μMol BV6 for 12 h and were washed before being labeled and introduced to CIK cells in experiments.

Next, these different pre-treated target cells were cultured on U-bottomed-96-well culture plates (NUNC, Langenselbold, Germany). CIK cells were added at an effector to target cell (E:T) ratio of 20:1. After another period of 3 h, 20 μL of supernatant was collected out of each well and introduced to 200 μL europium solution (Europium, Perkin Elmer, Turku, Finland). Fluorescence data were recorded using a time resolved fluorometer (1420-018 Victor, Perkin Elmer, Waltham, MA, USA). The measured signal correlated with the amount of destroyed cells. Maximum release of BATDA was obtained by incubating target cells with 4% triton (Triton X-100, Sigma-Aldrich, Munich, Germany). Target cells without effector cells were used as negative control (spontaneous release). The percentage of specific cytolysis was calculated as experimental release minus spontaneous release divided through maximum release minus spontaneous release of target cells multiplied by 100.

### Flow cytometry

Malignant and non-malignant target cells THP-1 and PMNCs were incubated for 4 h with 2.5 and/or 10 μMol BV6 followed by co-incubation with CIK cells at an E:T ratio of 5:1 for 3 h. BV6 pre-treated target cells and CIK cells alone served as controls. Cell suspensions were then stained using FITC Annexin V Apoptosis Detection Kit with 7AAD (Bio Legend, San Diego, CA, USA) according to the manufacturer’s instructions. Anti-human CD3-PC7, CD25-PE, and CD33-PE (Beckman Coulter, Krefeld, Germany) as well as forward scatter were used to differentiate between CD33^+^ THP-1, CD3^±^CD25^+^ CD33^±^ CIK cells and CD3^±^CD25^±^ PMNCs by flow cytometry (FC500 Beckman Coulter). Percentages of Annexin V-positive apoptotic and 7AAD-positive necrotic cells are shown. Cytotoxicity results included Annexin V-positive and/or 7AAD-positive cells.

### Immunofluorescent staining and confocal microscopy

Target cells were grown together with CIK cells in chamber slides. Cells were fixed with 4% paraformaldehyde, permeabilized with 0.2% triton X-100 for 30 min, and intracellularly stained with caspase-3 phycoerythrin (PE)-labeled for 30 min at room temperature. To distinguish between CIK and RH30 cells, CIK cells were stained with fluorescein isothiocyanate (FITC)-labeled anti-CD45 antibody for 30 min at room temperature. Images were obtained using a confocal microscope. Images were computer processed by Zeiss LSM Image Browser.

### Western blot analysis

BV6-mediated killing mechanism was analyzed in co-cultures of tumor and CIK cells. TE671 (TE) cells that were efficiently killed by combination therapy and grown in adherent cultures allowing a segregation of effector and target cells by several washing steps were used for analyses. Analyzed samples included TE and CIK cells alone, TE and CIK cells pre-incubated with 2.5 μMol BV6 for 4 h, TE cells co-cultured with CIK cells at an E:T ratio of 20:1 for 3 h, and TE cells pre-incubated with 2.5 μMol BV6 for 4 h followed by co-culture with CIK cells at an E:T ratio of 20:1 for 3 h. Co-cultures of TE and CIK cells were washed for the removal of CIK cells before TE cells were analyzed by western blot analysis. Western blot analysis was performed as described previously using the following antibodies: mouse anti-caspase-8 (1:1000; Alexis Biochemicals, Gruenberg, Germany), rabbit anti-Bid, rabbit anti-caspase-3, and rabbit anti-caspase-9 (1:1000; Cell Signaling, Beverly, MA, USA). Mouse anti-β-actin (1:10000; Sigma) was used as loading control (37). Goat anti-mouse IgG and goat anti-rabbit IgG conjugated to horseradish peroxidase (1:5000; Santa Cruz Biotechnology, Santa Cruz, CA, USA) were used as secondary antibodies. Enhanced chemiluminescence was used for detection (Amersham Bioscience, Freiburg, Germany).

### Inhibition of caspase activation by zVAD

BV6 and CIK cell-mediated cytotoxicity mechanism was analyzed by europium release assay against TE671 cells after 4 h pre-incubation with 2.5 μMol BV6. To analyze whether BV6 and CIK cell treatment induces apoptosis by caspase activation, 50 mM zVAD was added. zVAD is a cell-permeant pan caspase inhibitor that irreversibly binds to the catalytic site of caspase proteases and inhibits induction of apoptosis by the caspase cascade.

### Statistical analysis

Statistical significance was assessed by two-sided Student’s t-test using PRISM 6. Results from independent experiments are shown as mean with standard error of the mean (SEM).

## Results

### Cytotoxicity of CIK cells against tumor cells in the presence of BV6

**Figure 1:**
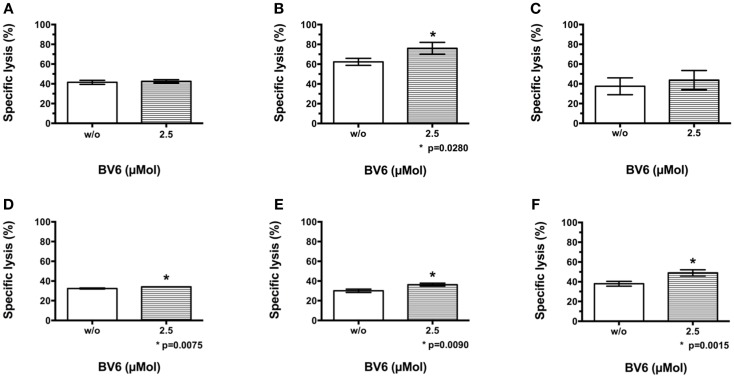
**CIK cell-mediated killing of malignant cells in the presence of BV6**. The potential of BV6 to increase CIK cell-mediated killing of hematological (H9, T cell lymphoma **(A)**; THP-1, subtype M4 acute myeloid leukemia **(B)**; Tanoue, precursor-B acute lymphoblastic leukemia **(C)**) and solid tumor targets (RH1, Ewing sarcoma **(D)**; RH30, alveolar rhabdomyosarcoma **(E)**; TE671, embryonal rhabdomyosarcoma **(F)**) was analyzed by europium release assay. Therefore, target cells were incubated with 2.5 μMol BV6 for 4 h or remained untreated. Thereafter, CIK cells were added for 3 h at an E:T ratio of 20:1. Results of at least three independent experiments are shown as mean with standard error of mean (mean ± SEM). Treatment with CIK cells alone served as standard control. Combination treatment of CIK cells and BV6 resulted in slightly increased lysis of targets cells in the presence of 2.5 μMol BV6.

Europium release assay was performed to evaluate the effect of BV6 on CIK cell-mediated killing of tumor targets. Thereafter, CIK cells were added for 3 h at an E:T ratio of 20:1. Europium release measurements confirmed CIK cell-mediated target cell killing in the presence of BV6 (Figure 1). Combination treatment of CIK cells and BV6 was referred to standard control without BV6 and resulted in moderately increased lysis of targets cells in the presence of 2.5 μMol BV6 (Figure 1: H9, (A); THP-1, (B); Tanoue, (C); RH1, (D); RH30, (E); TE671, (F)).

### Cytotoxicity of CIK cells against non-malignant PMNCs in the presence of BV6

**Figure 2:**
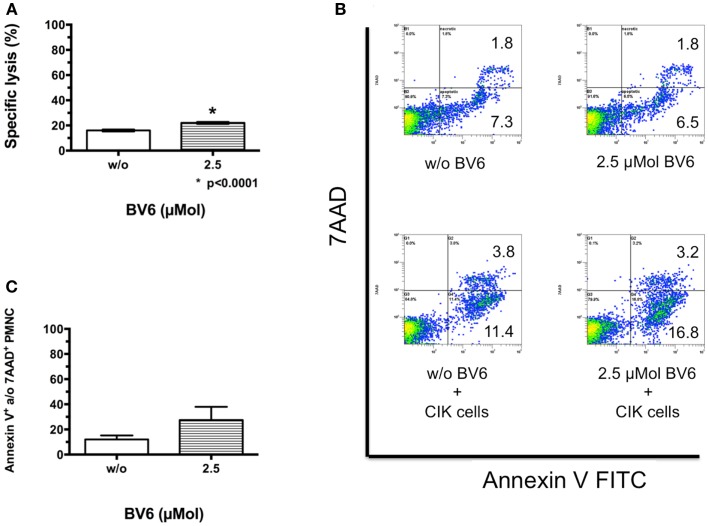
**CIK cell-mediated killing of non-malignant cells in the presence of BV6**. Cytotoxic potential of CIK cells combined with BV6 was analyzed against non-malignant cells like peripheral blood mononuclear cells (PMNCs, E:T ratio 20:1) by europium release assay **(A)**. Analysis of four independent experiments demonstrated as means with standard error of mean (mean ± SEM) revealed that combination treatment significantly increased CIK cell-mediated killing of PMNCs. However, cytotoxicity against non-malignant targets remained relatively low. Accordingly, PMNCs were treated with 2.5 μMol BV6 for 4 h or remained untreated and were incubated with CIK cells at an E:T ratio of 5:1 for 3 h. For analysis of apoptotic and necrotic cells, cell suspensions were stained with Annexin V FITC and 7AAD, respectively. PMNCs were further identified by forward scatter and excluding CD25-expressing larger CIK cells. One representative example showed that compared to untreated controls PMNCs were not affected by BV6, but by CIK cell treatment and furthermore by combination therapy **(B)**. Cytotoxicity data (Annexin V- and/or 7AAD-positive PMNCs) from three independent experiments show as mean ± SEM indicated that BV6 and CIK cell treatment in combination was also toxic for normal cells **(C)**.

To mimic cytotoxic potential of combination therapy of CIK cells and BV6 against non-malignant cells like PMNCs, the latter were freshly isolated by Ficoll^®^ density centrifugation, labeled, treated in part with BV6, and used for analysis in europium release assay. Combination treatment significantly increased CIK cell-mediated killing of PMNCs (mean ± SEM: w/o BV6, 16.0 ± 0.4%; 2.5 μMol BV6, 22.0 ± 0.4%, *p* < 0.0001, (*n* = 4); Figure 2A).

This analysis was also performed by flow cytometry as cytolysis of non-malignant cells may limit applicability of combination therapy. Therefore, PMNCs treated with 2.5 μMol BV6 and untreated PMNCs were incubated with CIK cells at an E:T ratio of 5:1. For analysis of apoptotic and necrotic cells, cell suspensions were stained with Annexin V FITC and 7AAD, respectively. PMNCs were identified by forward scatter and CD25-expression and excluding CD25-expressing larger CIK cells. Compared to untreated controls (apoptotic cells, 7.3%; necrotic cells, 1.8%), PMNCs were not affected by BV6 treatment alone (apoptotic cells, 6.5%; necrotic cells, 1.8%), but were impaired by CIK cell treatment (apoptotic cells, 11.4%; necrotic cells, 3.8%) and furthermore by combination therapy (apoptotic cells, 16.8%; necrotic cells, 3.2%) shown by one representative example (Figure 2B). Flow cytometry results of independent experiments showed that combination treatment increased CIK cell cytotoxicity toward PMNCs, but differences were not significant (mean ± SEM of Annexin V- and/or 7AAD-positive PMNCs: w/o BV6, 12.1 ± 3.2%; 2.5 μMol BV6, 27.5 ± 7.5%, (n = 3); Figure 2C).

### CIK cell cytotoxicity against tumor cells after pre-treatment and removal of BV6

**Figure 3:**
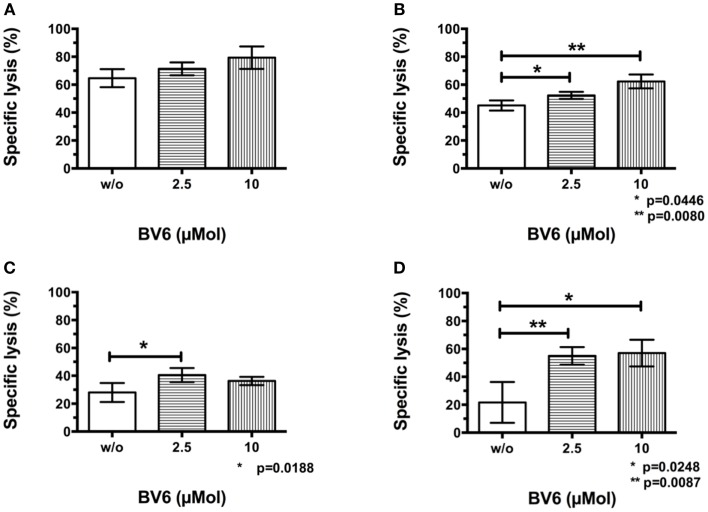
**CIK cell cytotoxicity against tumor cells after pre-treatment and removal of BV6**. Results of at least three independent experiments performed by europium release assay and shown as means with standard error of mean (mean ± SEM) indicated that removal of BV6 after a target cell pre-incubation period of 4 h increased CIK cell-mediated killing of hematological and solid malignancies like THP-1 **(A)** and RH30 cells **(B)** in a dose-dependent manner. Furthermore, europium release measurements were performed after extending BV6 sensitization period from 4 to 12 h to increase CIK cell-mediated cytotoxicity towards more resistant target cells like RH30 **(C)** and TE671 cell lines **(D)**. Cytotoxicity results showed that CIK cell-mediated killing of RH30 and TE671 cell lines was significantly but dose-independently increased, but there was no further enhancement compared to four-hour per-incubation of target cells with BV6.

To analyze whether target cell sensitization by BV6 was increasable, selected hematological (THP-1, Figure 3A) and solid malignancies (RH30, Figure 3B) were pre-treated with increasing concentrations of BV6 for 4 h and were washed before being incubated with CIK cells in the europium release assay. Sequential use of BV6 and CIK cells such that the SMAC mimetic effect is restricted to the targets, increased CIK cell-mediated killing of RH30 and THP-1 cells in a dose-dependent manner (THP-1, mean ± SEM: w/o BV6, 64.7 ± 3.7%; 2.5 μMol BV6, 71.3 ± 2.7%; 10 μMol BV6, 79.3 ± 4.7%, n.s. (*n* = 3), Figure 3A; RH30, w/o BV6, 45.0 ± 2.1%; 2.5 μMol BV6, 52.3 ± 1.5%, *p* = 0.0446; 10 μMol BV6, 62.3 ± 2.8%, *p* = 0.0080, (*n* = 3), Figure 3B). Hereby, maximum cell lysis was achieved after target cell pre-treatment with 10 μMol BV6.

In addition, relatively resistant target cell lines like RH30 (Figure 3C) and TE671 cells (Figure 3D) were incubated with BV6 for 12 h, washed and introduced to CIK cells in the europium release assay. Extended pre-incubation period also resulted in significantly increased CIK cell-mediated killing of RH30 and TE671 cells, but increase of cytotoxicity was not dose-dependent and was not enhanced compared to 4 h target cell pre-incubation period (RH30, w/o BV6, 28.0 ± 3.1%; 2.5 μMol BV6, 40.5 ± 2.5%, *p* = 0.0188; 10 μMol BV6, 36.3 ± 1.5%; (*n* = 4), Figure 3C; TE671, w/o BV6, 21.7 ± 8.5%; 2.5 μMol BV6, 55.0 ± 3.1%, *p* = 0.0087; 10 μMol BV6, 57.0 ± 5.5%, *p* = 0.0248; (*n* = 3), Figure 3D).

### CIK cell cytotoxicity against tumor cells in the presence of dose escalated BV6

**Figure 4:**
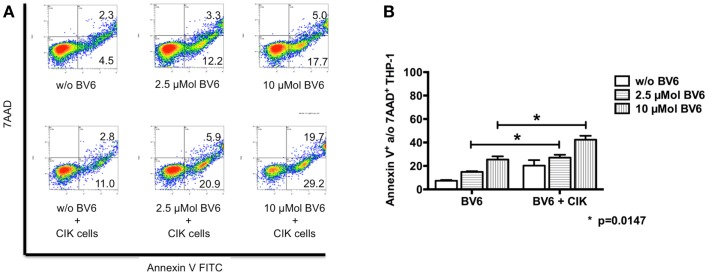
**CIK cell-mediated cytotoxicity after dose escalation of BV6 pre-treatment**. Cytotoxic effects of both, increasing doses of BV6 and CIK cells were analyzed by flow cytometry. One representative example is shown **(A)**. Hereby, THP-1 cells were treated with 2.5 or 10 μMol BV6 for 4 h and were incubated with CIK cells at an E:T ratio of 5:1 for another 3 h. THP-1 and CIK cells were differentiated by forward scatter and CD33-expression. Analysis of apoptotic (Annexin V FITC) and necrotic cells (7AAD) showed that combination therapy increased cytotoxicity against CD33-expressing larger THP-1 cells. Cytotoxicity data (Annexin V- and/or 7AAD-positive THP-1 cells) from three independent experiments shown as mean ± SEM demonstrated that BV6 and CIK cell treatment in combination significantly increased cytolysis of THP-1 cells **(B)**.

Dose escalating effects of BV6 regarding tumor cell sensitization toward CIK cell-mediated killing was also analyzed by flow cytometry. Therefore, THP-1 cells with BV6 pre-treatment were incubated with CIK cells at an E:T ratio of 5:1. THP-1 cells alone and THP-1 cells with BV6 or CIK cell treatment alone served as controls. Flow cytometric analysis of apoptotic (Annexin V-positive) and necrotic (7AAD-positive) THP-1 cells (shown by one represented example, Figure 4A) demonstrated that cytotoxic effects of BV6 (THP-1, mean ± SEM: w/o BV6, 7.4 ± 0.6%; 2.5 μMol BV6, 15.0 ± 0.6%; 10 μMol BV6, 25.5 ± 2.8%) and CIK cells (THP-1, mean ± SEM: 20.3 ± 4.6%) alone were moderate, but significantly increased in a dose-dependent manner after combining BV6 and CIK cell treatment (THP-1, mean ± SEM: 2.5 μMol BV6 + CIK cells, 27.2 ± 2.4%; 10 μMol BV6 + CIK cells, 42.4 ± 3.3%, Figure 4B).

### Influences of BV6 and target cells on CIK cells

**Figure 5:**
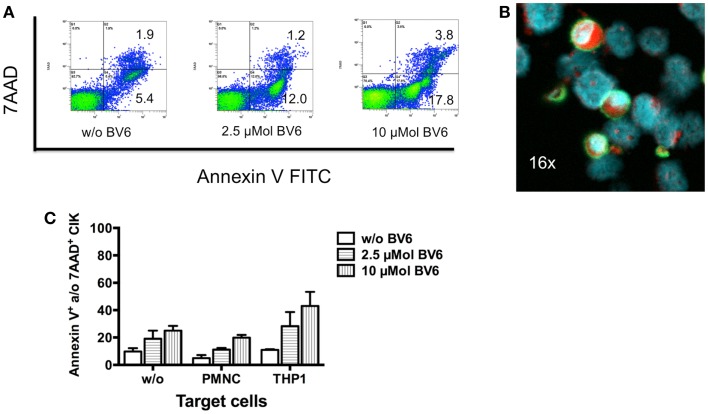
**Induction of apoptosis in CIK cells by SMAC mimetic or killing of target cells**. **(A)** The cytotoxic potential of 2.5 and 10 μMol of the SMAC mimetic BV6 on CIK cells was analyzed by flow cytometry. One representative example showed that CIK cells were affected by increasing doses of BV6 indicated by escalating levels of apoptotic (Annexin V) and necrotic (7AAD) CIK cells **(A)**. After 3 h of co-incubation with alveolar rhabdomyosarcoma cell line RH30 CIK cells adhered to tumor cells, which was shown by one representative example using fluorescence microscope analysis (magnification; 16×). CIK cells were visualized by surface staining with fluorescein isothiocyanate (FITC)-labeled anti-CD45 antibody (green fluorescence). Nuclei of all cells were stained with DAPI (blue fluorescence). As a sign of ongoing apoptosis, CIK cells being in contact with tumor cells and tumor cells both showed intracellular expression of caspase-3 stained with phycoerythrin (PE) (red fluorescence) **(B)**. Cytotoxicity of BV6 on CIK cells alone and in the presence of non-malignant cells (PMNC) and malignant (THP-1) cells (E:T ratio, 5:1) was analyzed by flow cytometry. CIK cells within co-cultures were identified by forward scatter and CD3-, CD25-, and CD33-expression. Cytotoxicity results (mean ± SEM of Annexin V- and/or 7AAD-positive CIK cells) from three independent experiments showed that cytolysis of CIK cells increased with escalating doses of BV6 especially against malignant targets **(C)**.

To analyze whether BV6 also influences CIK cells and thereby their cytotoxic potential, CIK cells were analyzed after four-hour incubation with 2.5 and 10 μMol BV6. Results measured by flow cytometry showed that CIK cells were affected by BV6 in a dose-dependent manner (w/o BV6, apoptotic cells, 5.4%; necrotic cells, 1.9%; 2.5 μMol BV6, apoptotic cells, 12.0%; necrotic cells, 1.2%; 10 μMol BV6, apoptotic cells, 17.8%; necrotic cells, 3.8%) (Figure 5A). Next we analyzed whether killing of target cells induced apoptosis in CIK cells. To this end, CIK cells were co-incubation with alveolar rhabdomyosarcoma cell line RH30. CIK cells were stained with FITC-labeled anti-CD45 antibody. Induction of apoptosis was shown by expression of caspase-3 stained with PE and visualized by fluorescence microscope analysis. As a sign of ongoing apoptosis, CIK cells being in contact with tumor cells and tumor cells both expressed caspase-3 (Figure 5B). Cytotoxicity of BV6 on CIK cells alone or in the presence of malignant (THP-1) and non-malignant cells (PMNC) was analyzed by flow cytometry. Cytotoxicity results showed that cytolysis of CIK cells increased with killing of malignant targets and escalating doses of BV6 (Figure 5C).

### CIK cell-mediated killing of tumor cells by BV6 involves the caspase cascade

**Figure 6:**
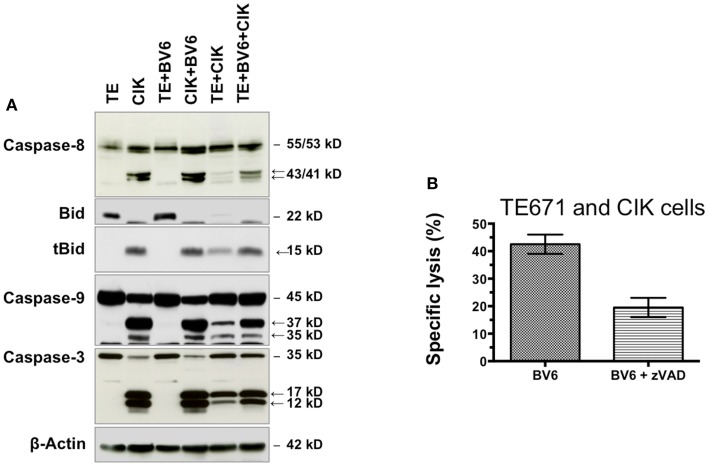
**BV6 and CIK cells reactivate apoptosis in TE671 cells**. TE671 cells (TE) were treated for 4 h with 2.5 μM SMAC mimetic BV6 before BV6 was removed and CIK cells were added for 3 h (lane 6). TE and CIK cells alone (lanes 1 and 2), and after 4 h pre-incubation with 2.5 μM BV6 (lines 3 and 4), as well as TE cells after 3 h treatment with CIK cells (lane 5) served as controls. CIK cells within samples of lanes 5 and 6 were removed, before western blot analysis was performed. Caspase activation and Bid cleavage were analyzed. Cleavage fragments are indicated by arrows. β-Actin served as loading control. CIK cell treatment without (lane 5) or particularly with SMAC mimetic BV6 (lane 6) increased TE killing by activation of the caspase cascade. This was evident from the increased cleavage of caspase-8, -3, and -9 into active cleavage fragments in the presence of BV6 and CIK cells, that is, caspase-8 into active p43 and p41 fragments, caspase-3 into active p17 and p12 fragments and caspase-9 into active p37 and p35 fragments. Moreover, Bid was predominately cleaved into tBid, the activated form of Bid, upon BV6 and CIK cell treatment **(A)**. The addition of zVAD a pan caspase inhibitor reduced CIK cell-mediated killing of TE671 cells by 50% demonstrating that BV6 sensitized target cells towards cytotoxicity of CIK cells by caspase activation **(B)**.

Western blot analysis was used to identify underlying killing mechanism of combination therapy (Figure 6A). Compared to TE671 (TE) cells (lane 1), CIK cells (lane 2) showed a high activation of apoptotic pathway. Four-hour pre-incubation with BV6 had no additional effects on TE (lane 3) and CIK cells (lane 4) alone. CIK cell treatment alone (lane 5) or in combination with SMAC mimetic BV6 (lane 6) increased TE killing by activation of the caspase cascade (Figure 6A, in lane 5 and 6 TE results after removal of CIK cells are shown). This was evident from the increased cleavage of caspase-8, -3, and -9 into active cleavage fragments in the presence of BV6 and CIK cells, that is, caspase-8 into active p43 and p41 fragments, caspase-3 into active p17 and p12 fragments and caspase-9 into active p37 and p35 fragments. Moreover, Bid was predominately cleaved into tBid, the activated form of Bid, upon BV6 and CIK cell treatment (Figure 6A). Addition of zVAD a caspase inhibitor reduced killing of TE671 cells by 50% (Figure 6B). Collectively, these data demonstrate that BV6 increases susceptibility of TE671 cells to CIK cell-mediated cytolysis by triggering caspases activation and apoptosis.

## Discussion

Resistance of tumor cells still remains an unsolved problem in high-risk cancers after allogeneic HSCT. Tumor cells may even survive additional cell therapeutic strategies for augmentation of allogeneic effects post-transplant. Sequential therapy with apoptosis-sensitizing agents was previously shown for chemotherapy, radiation, HDAC inhibitors, JAK/STAT inhibitors, proteasome inhibitors, or tyrosine kinase inhibitors, but not CIK cells (38–46). Improved therapeutic options may therefore include non-MHC restricted CIK cell infusions combined with targeted cancer therapies.

Due to the fact that IAP expression or function is deregulated in many refractory cancers, several IAP-targeting agents including SMAC mimetics are currently being tested *in vitro* and *in vivo* in early clinical trials (21–26, 47–51). It was found that SMAC mimetics allow sensitization of tumor cells for apoptosis induction by releasing caspases from the inhibitory interaction with IAP proteins, particular XIAP (49, 52) and that SMAC mimetics have complex effects on NFκB and TNF signaling (23, 26, 29–31, 51, 53, 54). But, due to the importance of NFκB in immune cells, SMAC mimetics might also modulate immune cell functions and therefore influence cell-based combination therapies, e.g., by modulating NFκB signaling in immune cells. In this study, we analyzed the cytotoxic effects of CIK cells against tumor cells in the presence of the bivalent SMAC mimetic BV6.

Our results showed that BV6 sensitization of hematological and solid malignancies, i.e., H9, THP-1, Tanoue, RH1, RH30, and TE671 cell lines toward CIK cell-mediated killing was limited. To analyze and further increase the impact of BV6 treatment on target cell sensitization, target cells were incubated with escalating doses of BV6 and incubation period was extended from 4 to 12 h before BV6 was removed and CIK cells were added for cytotoxicity analysis. In fact, when used sequentially increased dosage of BV6 correlated with the greater BV6 sensitivity of target cell lines toward CIK cell-mediated killing. However, cytotoxicity was not further increasable by extending BV6 incubation period. Monitoring cleavage of caspases by western blot analysis revealed that CIK cells without or with BV6 increased activation of caspases-3, -8, and -9 and cleavage of Bid to tBid. The truncated form of Bid (i.e., tBid) links the extrinsic apoptotic pathway and the intrinsic (mitochondrial) apoptotic pathway by triggering mitochondrial outer membrane permeabilization (MOMP).

However, BV6 both sensitized target cells for killing and had an inhibitory effect on effector cells. For assessment of BV6 effects on CIK cells, which may limit applicability of combination therapy, CIK cells were treated with increasing doses of BV6. Remarkably, not only BV6 treatment, but also the presence of target cells alone, induced apoptosis in CIK cells. Accordingly, Nishimura et al. (55) demonstrated high percentages of early apoptotic cells in mice transplanted with CIK cells alone, suggesting that CIK cells *per se* demonstrated limited life span and increased susceptibility to apoptosis. Our results confirmed high expression levels of active caspase-3 fragments as a sign of activated apoptosis pathways in CIK cells being in contact with tumor cells. Interestingly, increased caspase-3-like activity has previously been detected in non-apoptotic T cells demonstrating that caspase-3-activation does not necessarily lead to apoptosis (56). Alternatively, CIK cells may undergo apoptosis during killing of tumor cells. Therefore the relevance of caspase activation in CIK cells remains to be explored in future studies.

Altogether, our results showed that CIK cells were affected by BV6. However, despite being affected as well by BV6, adding CIK cells to co-cultures of BV6 and tumor cells significantly increased cytotoxicity against malignant cells compared to BV6 or CIK cell treatment alone. This may allow clinical applicability of combination therapy. However, further pre-clinical analyzes will be mandatory to optimize BV6 dosage and time interval between BV6 and CIK cell applications. Furthermore, serial CIK cell infusions may be considered in this context. Accordingly, effects of SMAC mimetic BV6 on conventional immune cells were analyzed by Muller-Sienerth et al. (57). Considering the role of NFκB transcription factors in the immune system, they found that BV6-induced apoptotic and necrotic cell death in monocytes while T cells, dendritic cells, and macrophages were largely protected against BV6-induced cell death.

Of note, our results also demonstrated BV6-mediated sensitization of non-malignant cells like PMNCs toward CIK cell-mediated killing, which may increase risk for graft versus host disease or graft failure and therefore may complicate the development of tumor therapeutic concepts based on CIK cell infusions combined with BV6 cancer therapies. However, absolute cytotoxicity values against PMNCs ranged between 16.0 ± 0.40% and 22.0 ± 0.4% while specific lysis of malignant cells reached a maximum of 62.3 ± 2.0% and 76.0 ± 3.5% without or after addition of 2.5 μMol BV6, respectively. This difference may hint toward a more selective killing of malignant cells while almost sparing non-malignant cells. Moreover our clinical data showed that even in the haploidentical stem cell transplantation setting, alloreactive potential of CIK cells remained low. Here, serial infusions of 1 × 10^8^ CD3^+^CD56^−^ CIK cells/kg recipient body weight generated from the original haploidentical stem cell donor for treatment of relapse post-transplant were well tolerated and no signs of acute graft versus host disease occurred (GvHD) (58).

Moreover, due to the diverse character of CIK cells it remained unclear, which effector cell population was supported most by the administration of SMAC mimetic BV6. Hence, more specific cell therapy approaches such as CAR T cells, TCR-transduced T cells, or specific T cell clones would have been more applicable in combination with BV6. Further analyses may also address the question, if the effector cell inhibition refers to BV6 *per se*, or SMAC mimetics in general. Another IAP, i.e., Birinapant (TL32711) a bivalent SMAC mimetic that recently afforded synergistic cytotoxicity of chemotherapeutic agents and displayed significant antitumor activity at well-tolerated doses in murine xenograft models could also be analyzed in context of cell therapy-based combination strategies (35).

In conclusion, CIK cell-mediated cytotoxicity by target cell pre-treatment with the IAP BV6 was limited as CIK cells were also affected by BV6. However, cytotoxic effects were dose- and target cell-dependent and increased especially when BV6 and CIK cells were used sequentially compared to BV6 or CIK cell treatment alone. Altogether, the combination strategy of cellular therapies and IAP may represent a treatment approach for resistant tumor targets, which still warrants further investigation in pre-clinical *in vitro* and *in vivo* models.

## Author Contributions

Eva Rettinger, Andreas Glatthaar, Behnaz Ahangarian Abhari, Sarah Oelsner, Verena Pfirrmann, Sabine Huenecke, and Peter Bader acquired, analysed, and helped interpret data. Selim Kuçi, Hermann Kreyenberg, Andre M. Willasch, Thomas Klingebiel, and Simone Fulda participated in and contributed to the acquisition and interpretation of data. All authors reviewed and approved the final version of the manuscript.

## Conflict of Interest Statement

The authors declare that the research was conducted in the absence of any commercial or financial relationships that could be construed as a potential conflict of interest.

## References

[1] Rettinger E Willasch AM Kreyenberg H Borkhardt A Holter W Kremens B Preemptive immunotherapy in childhood acute myeloid leukemia for patients showing evidence of mixed chimerism after allogeneic stem cell transplantation. Blood (2011) 118:5681–8 10.1182/blood-2011-04-348805 21948300

[2] Porter DL Levine BL Kalos M Bagg A June CH Chimeric antigen receptor-modified T cells in chronic lymphoid leukemia. N Engl J Med (2011) 365:725–33 10.1056/NEJMoa1103849 21830940 PMC3387277

[3] Savoldo B Ramos CA Liu E Mims MP Keating MJ Carrum G CD28 costimulation improves expansion and persistence of chimeric antigen receptor-modified T cells in lymphoma patients. J Clin Invest (2011) 121:1822–6 10.1172/JCI46110 21540550 PMC3083795

[4] Morgan RA Dudley ME Wunderlich JR Hughes MS Yang JC Sherry RM Cancer regression in patients after transfer of genetically engineered lymphocytes. Science (2006) 314:126–9 10.1126/science.1129003 16946036 PMC2267026

[5] Kronig H Hofer K Conrad H Guilaume P Muller J Schiemann M Allorestricted T lymphocytes with a high avidity T-cell receptor towards NY-ESO-1 have potent anti-tumor activity. Int J Cancer (2009) 125:649–55 10.1002/ijc.24414 19444908

[6] Peggs KS Verfuerth S Pizzey A Khan N Guiver M Moss PA Adoptive cellular therapy for early cytomegalovirus infection after allogeneic stem-cell transplantation with virus-specific T-cell lines. Lancet (2003) 362:1375–7 10.1016/S0140-6736(03)14634-X 14585640

[7] Heslop HE Slobod KS Pule MA Hale GA Rousseau A Smith CA Long-term outcome of EBV-specific T-cell infusions to prevent or treat EBV-related lymphoproliferative disease in transplant recipients. Blood (2010) 115:925–35 10.1182/blood-2009-08-239186 19880495 PMC2817637

[8] Schmidt-Wolf GD Negrin RS Schmidt-Wolf IG Activated T cells and cytokine-induced CD3+CD56+ killer cells. Ann Hematol (1997) 74:51–6 10.1007/s002770050257 9063373

[9] Sangiolo D Martinuzzi E Todorovic M Vitaggio K Vallario A Jordaney N Alloreactivity and anti-tumor activity segregate within two distinct subsets of cytokine-induced killer (CIK) cells: implications for their infusion across major HLA barriers. Int Immunol (2008) 20:841–8 10.1093/intimm/dxn042 18469328

[10] Franceschetti M Pievani A Borleri G Vago L Fleischhauer K Golay J Cytokine-induced killer cells are terminally differentiated activated CD8 cytotoxic T-EMRA lymphocytes. Exp Hematol (2009) 37(616–628):e612 10.1016/j.exphem.2009.01.010 19375652

[11] Linn YC Lau SK Liu BH Ng LH Yong HX Hui KM Characterization of the recognition and functional heterogeneity exhibited by cytokine-induced killer cell subsets against acute myeloid leukaemia target cell. Immunology (2009) 126:423–35 10.1111/j.1365-2567.2008.02910.x 18778291 PMC2669823

[12] Sangiolo D Mesiano G Carnevale-Schianca F Piacibello W Aglietta M Cignetti A Cytokine induced killer cells as adoptive immunotherapy strategy to augment graft versus tumor after hematopoietic cell transplantation. Expert Opin Biol Ther (2009) 9:831–40 10.1517/14712590903005552 19463075

[13] Kuci S Rettinger E Voss B Weber G Stais M Kreyenberg H Efficient lysis of rhabdomyosarcoma cells by cytokine-induced killer cells: implications for adoptive immunotherapy after allogeneic stem cell transplantation. Haematologica (2010) 95:1579–86 10.3324/haematol.2009.019885 20378565 PMC2930961

[14] Linn YC Hui KM Cytokine-induced NK-like T cells: from bench to bedside. J Biomed Biotechnol (2010) 2010:435745 10.1155/2010/435745 20368995 PMC2847766

[15] Pievani A Borleri G Pende D Moretta L Rambaldi A Golay J Dual-functional capability of CD3+CD56+ CIK cells, a T-cell subset that acquires NK function and retains TCR-mediated specific cytotoxicity. Blood (2011) 118:3301–10 10.1182/blood-2011-02-336321 21821703

[16] Sangiolo D Cytokine induced killer cells as promising immunotherapy for solid tumors. J Cancer (2011) 2:363–8 10.7150/jca.2.363 21716717 PMC3119405

[17] Rettinger E Kuci S Naumann I Becker P Kreyenberg H Anzaghe M The cytotoxic potential of interleukin-15-stimulated cytokine-induced killer cells against leukemia cells. Cytotherapy (2012) 14:91–103 10.3109/14653249.2011.613931 21973023

[18] Introna M Borleri G Conti E Franceschetti M Barbui AM Broady R Repeated infusions of donor-derived cytokine-induced killer cells in patients relapsing after allogeneic stem cell transplantation: a phase I study. Haematologica (2007) 92:952–9 10.3324/haematol.11132 17606446

[19] Laport GG Sheehan K Baker J Armstrong R Wong RM Lowsky R Adoptive immunotherapy with cytokine-induced killer cells for patients with relapsed hematologic malignancies after allogeneic hematopoietic cell transplantation. Biol Blood Marrow Transplant (2011) 17:1679–87 10.1016/j.bbmt.2011.05.012 21664472 PMC3175285

[20] Linn YC Niam M Chu S Choong A Yong HX Heng KK The anti-tumour activity of allogeneic cytokine-induced killer cells in patients who relapse after allogeneic transplant for haematological malignancies. Bone Marrow Transplant (2012) 47:957–66 10.1038/bmt.2011.202 21986635

[21] Fulda S Inhibitor of apoptosis proteins in hematological malignancies. Leukemia (2009) 23:467–76 10.1038/leu.2008.329 19039324

[22] Gyrd-Hansen M Meier P IAPs: from caspase inhibitors to modulators of NF-kappaB, inflammation and cancer. Nat Rev Cancer (2010) 10:561–74 10.1038/nrc2889 20651737

[23] Varfolomeev E Blankenship JW Wayson SM Fedorova AV Kayagaki N Garg P IAP antagonists induce autoubiquitination of c-IAPs, NF-kappaB activation, and TNFalpha-dependent apoptosis. Cell (2007) 131:669–81 10.1016/j.cell.2007.10.030 18022362

[24] Wang X The expanding role of mitochondria in apoptosis. Genes Dev (2001) 15:2922–33 11711427

[25] Srinivasula SM Hegde R Saleh A Datta P Shiozaki E Chai J A conserved XIAP-interaction motif in caspase-9 and Smac/DIABLO regulates caspase activity and apoptosis. Nature (2001) 410:112–6 10.1038/35065125 11242052

[26] Vince JE Wong WW Khan N Feltham R Chau D Ahmed AU IAP antagonists target cIAP1 to induce TNFalpha-dependent apoptosis. Cell (2007) 131:682–93 10.1016/j.cell.2007.10.037 18022363

[27] Vallabhapurapu S Matsuzawa A Zhang W Tseng PH Keats JJ Wang H Nonredundant and complementary functions of TRAF2 and TRAF3 in a ubiquitination cascade that activates NIK-dependent alternative NF-kappaB signaling. Nat Immunol (2008) 9:1364–70 10.1038/ni.1678 18997792 PMC2671996

[28] Zarnegar B Yamazaki S He JQ Cheng G Control of canonical NF-kappaB activation through the NIK-IKK complex pathway. Proc Natl Acad Sci U S A (2008) 105:3503–8 10.1073/pnas.0707959105 18292232 PMC2265190

[29] Zarnegar BJ Wang Y Mahoney DJ Dempsey PW Cheung HH He J Noncanonical NF-kappaB activation requires coordinated assembly of a regulatory complex of the adaptors cIAP1, cIAP2, TRAF2 and TRAF3 and the kinase NIK. Nat Immunol (2008) 9:1371–8 10.1038/ni.1676 18997794 PMC2676931

[30] Mahoney DJ Cheung HH Mrad RL Plenchette S Simard C Enwere E Both cIAP1 and cIAP2 regulate TNFalpha-mediated NF-kappaB activation. Proc Natl Acad Sci U S A (2008) 105:11778–83 10.1073/pnas.0711122105 18697935 PMC2575330

[31] Varfolomeev E Goncharov T Fedorova AV Dynek JN Zobel K Deshayes K c-IAP1 and c-IAP2 are critical mediators of tumor necrosis factor alpha (TNFalpha)-induced NF-kappaB activation. J Biol Chem (2008) 283:24295–9 10.1074/jbc.C800128200 18621737 PMC3259840

[32] Haas TL Emmerich CH Gerlach B Schmukle AC Cordier SM Rieser E Recruitment of the linear ubiquitin chain assembly complex stabilizes the TNF-R1 signaling complex and is required for TNF-mediated gene induction. Mol Cell (2009) 36:831–44 10.1016/j.molcel.2009.10.013 20005846

[33] Dueber EC Schoeffler AJ Lingel A Elliott JM Fedorova AV Giannetti AM Antagonists induce a conformational change in cIAP1 that promotes autoubiquitination. Science (2011) 334:376–80 10.1126/science.1207862 22021857

[34] Fulda S Vucic D Targeting IAP proteins for therapeutic intervention in cancer. Nat Rev Drug Discov (2012) 11:109–24 10.1038/nrd3627 22293567

[35] Benetatos CA Mitsuuchi Y Burns JM Neiman EM Condon SM Yu G Birinapant (TL32711), a bivalent SMAC mimetic, targets TRAF2-associated cIAPs, abrogates TNF-induced NF-kappaB activation, and is active in patient-derived xenograft models. Mol Cancer Ther (2014) 13:867–79 10.1158/1535-7163.MCT-13-0798 24563541

[36] Benetatos CA Burns JM Borden EC Lindner D Mitsuuchi Y Mckinlay MA The Smac Mimetic Birinapant synergistically induces apoptosis in combination with type I interferons and GM-CSF. AARC Annual Meeting (2013) 73:Abstract 3336. 10.1158/1538-7445.AM2013-3336

[37] Fulda S Sieverts H Friesen C Herr I Debatin KM The CD95 (APO-1/Fas) system mediates drug-induced apoptosis in neuroblastoma cells. Cancer Res (1997) 57:3823–9 9288794

[38] Arnt CR Chiorean MV Heldebrant MP Gores GJ Kaufmann SH Synthetic Smac/DIABLO peptides enhance the effects of chemotherapeutic agents by binding XIAP and cIAP1 in situ. J Biol Chem (2002) 277:44236–43 10.1074/jbc.M207578200 12218061

[39] Mitsiades N Mitsiades CS Richardson PG Poulaki V Tai YT Chauhan D The proteasome inhibitor PS-341 potentiates sensitivity of multiple myeloma cells to conventional chemotherapeutic agents: therapeutic applications. Blood (2003) 101:2377–80 10.1182/blood-2002-06-1768 12424198

[40] Diaz-Rubio E New chemotherapeutic advances in pancreatic, colorectal, and gastric cancers. Oncologist (2004) 9:282–94 10.1634/theoncologist.9-3-282 15169983

[41] Dorai T Aggarwal BB Role of chemopreventive agents in cancer therapy. Cancer Lett (2004) 215:129–40 10.1016/j.canlet.2004.07.013 15488631

[42] Gschwind A Fischer OM Ullrich A The discovery of receptor tyrosine kinases: targets for cancer therapy. Nat Rev Cancer (2004) 4:361–70 10.1038/nrc1360 15122207

[43] Frew AJ Johnstone RW Bolden JE Enhancing the apoptotic and therapeutic effects of HDAC inhibitors. Cancer Lett (2009) 280:125–33 10.1016/j.canlet.2009.02.042 19359091

[44] Probst BL Liu L Ramesh V Li L Sun H Minna JD Smac mimetics increase cancer cell response to chemotherapeutics in a TNF-alpha-dependent manner. Cell Death Differ (2010) 17:1645–54 10.1038/cdd.2010.44 20431601 PMC3104849

[45] Berger R Jennewein C Marschall V Karl S Cristofanon S Wagner L NF-kappaB is required for Smac mimetic-mediated sensitization of glioblastoma cells for gamma-irradiation-induced apoptosis. Mol Cancer Ther (2011) 10:1867–75 10.1158/1535-7163.MCT-11-0218 21859841

[46] Greer RM Peyton M Larsen JE Girard L Xie Y Gazdar AF SMAC mimetic (JP1201) sensitizes non-small cell lung cancers to multiple chemotherapy agents in an IAP-dependent but TNF-alpha-independent manner. Cancer Res (2011) 71:7640–8 10.1158/0008-5472.CAN-10-3947 22049529 PMC3382117

[47] Petersen SL Wang L Yalcin-Chin A Li L Peyton M Minna J Autocrine TNFalpha signaling renders human cancer cells susceptible to Smac-mimetic-induced apoptosis. Cancer Cell (2007) 12:445–56 10.1016/j.ccr.2007.08.029 17996648 PMC3431210

[48] Gyrd-Hansen M Darding M Miasari M Santoro MM Zender L Xue W IAPs contain an evolutionarily conserved ubiquitin-binding domain that regulates NF-kappaB as well as cell survival and oncogenesis. Nat Cell Biol (2008) 10:1309–17 10.1038/ncb1789 18931663 PMC2818601

[49] Lacasse EC Mahoney DJ Cheung HH Plenchette S Baird S Korneluk RG IAP-targeted therapies for cancer. Oncogene (2008) 27:6252–75 10.1038/onc.2008.302 18931692

[50] Lu J Bai L Sun H Nikolovska-Coleska Z Mceachern D Qiu S SM-164: a novel, bivalent Smac mimetic that induces apoptosis and tumor regression by concurrent removal of the blockade of cIAP-1/2 and XIAP. Cancer Res (2008) 68:9384–93 10.1158/0008-5472.CAN-08-2655 19010913 PMC2784911

[51] Wang L Du F Wang X TNF-alpha induces two distinct caspase-8 activation pathways. Cell (2008) 133:693–703 10.1016/j.cell.2008.03.036 18485876

[52] Chen DJ Huerta S Smac mimetics as new cancer therapeutics. Anticancer Drugs (2009) 20:646–58 10.1097/CAD.0b013e32832ced78 19550293

[53] Wang CY Mayo MW Korneluk RG Goeddel DV Baldwin AS Jr NF-kappaB antiapoptosis: induction of TRAF1 and TRAF2 and c-IAP1 and c-IAP2 to suppress caspase-8 activation. Science (1998) 281:1680–3 10.1126/science.281.5383.1680 9733516

[54] Li L Thomas RM Suzuki H De Brabander JK Wang X Harran PG A small molecule Smac mimic potentiates TRAIL- and TNFalpha-mediated cell death. Science (2004) 305:1471–4 10.1126/science.1098231 15353805

[55] Nishimura R Baker J Beilhack A Zeiser R Olson JA Sega EI In vivo trafficking and survival of cytokine-induced killer cells resulting in minimal GVHD with retention of antitumor activity. Blood (2008) 112:2563–74 10.1182/blood-2007-06-092817 18565854 PMC2532819

[56] Wilhelm S Wagner H Hacker G Activation of caspase-3-like enzymes in non-apoptotic T cells. Eur J Immunol (1998) 28:891–900 10.1002/(SICI)1521-4141(199803)28:03<891::AID-IMMU891>3.0.CO;2-x 9541584

[57] Muller-Sienerth N Dietz L Holtz P Kapp M Grigoleit GU Schmuck C SMAC mimetic BV6 induces cell death in monocytes and maturation of monocyte-derived dendritic cells. PLoS One (2011) 6:e21556 10.1371/journal.pone.0021556 21738708 PMC3127953

[58] Rettinger E Bonig H Wehner S Lucchini G Willasch A Jarisch A Feasibility of IL-15-activated cytokine-induced killer cell infusions after haploidentical stem cell transplantation. Bone Marrow Transplant (2013) 48:1141–3 10.1038/bmt.2013.19 23474803

